# Book Review

**Published:** 1946

**Authors:** 


### Reviews of Books

**Endocrine Disorders in Childhood and Adolescence.** By H. S. Le Marquand, M.D., M.R.C.P., and F. W. H. Tozer, M.D., M.R.C.P. Pp. x., 298. Illustrated. London: Hodder & Stoughton. 1943. Price 15s.—The authors of this book have succeeded admirably in their aim of presenting the results of ten years' work in a general medical out-patient department on the investigation and treatment of endocrine disorders in the child and adolescent. There is a useful introductory description of the physiology of the endocrine glands, and the usual endocrine syndromes are then well classified and carefully described, stress being laid throughout on clinical diagnosis. The sections on treatment are of particular merit, being essentially practical, that on the treatment of the overweight child being worthy of special mention, although the authors appear to take a more optimistic view of pituitary whole gland in treatment than is warranted by the experience of most physicians. In subsequent editions one would hope to see fuller accounts of some of the newer and more highly potent endocrine preparations, such as those of gonadotrophic hormones and pitressin in oily solution. The subject matter is attractively presented, with many useful illustrations and tables. The book should be of great value to medical students and general practitioners.

**Infant Feeding in General Practice.** By J. V. Braithwaite, M.D., F.R.C.P. Second Edition. Pp. xii., 165. Bristol: John Wright & Sons Ltd. 1943. Price 7s. 6d.—In the second edition of this little book the author has again achieved his object of producing in simple form a comprehensive summary of this subject, for general practitioners essentially, but equally valuable for the student. The author, himself a general practitioner until just before the publication of this edition, stresses the practical aspect and the trivial difficulties encountered in practice, but, at the same time, gives sufficient theoretical knowledge to put his arguments on a sound basis. The section on the chemistry of infant nutrition is particularly welcome in this respect. The early chapters on breast-feeding are full of sound advice and free from dogmatic teaching, but the inference that the breast-fed child does not need extra vitamins is not in accord with modern teaching. It is also regrettable that more space has not been devoted to practical details of diet during the difficult weaning period. Altogether this may be considered a most useful pocket handbook which should help to dispel the mass of ignorance which often surrounds this important subject.

**Quarterly Review of Pædiatrics, Vol. I, No. 1.** Washington, D.C.: Washington Institute of Medicine. 1946. Annual subscription, 54s.—This new journal will be welcomed by all who endeavour to keep abreast of progress in Pædiatrics. The Editorial Board includes many eminent American Pædiatricians, which should be a guarantee of the high standard to be expected. Abstracts and references to current literature have been collected from world pædiatric literature and appear to be exceptionally comprehensive. The abstracts are well selected and written concisely, but with adequate detail, and useful editorial comments are added. The classification, in systems and special subjects, gives a comprehensive review of certain special subjects, but the reader might be helped by a list of cross references at the end of each section. As an example, the section on the Newborn does not include references to penicillin treatment or psychological investigations in this age group, these having been included under other special sections. This deficiency is only partially remedied by the careful subject index. In the American tradition, printing and general set-up is good.

**Aids to Medical Diagnosis.** By G. E. FREDERICK SUTTON, M.C., M.D., M.R.C.P. Sixth Edition. Pp. viii., 308. Illustrated. London: Bailliere, Tindall & Cox. 1946. Price 6s.—The Sixth Edition of *Aids to Medical Diagnosis* has been entrusted to Dr. Sutton, and this revision in no way falls short of the previous ones. Many of the sections have been completely rewritten and new work has been very adequately included. In particular, the chapter dealing with "Diseases of the Heart and Bloodvessels" deserves praise for its comprehensiveness and clarity of writing. "Diseases of the Blood" is another up-to-date chapter, the references to the Rh factor are brief and serve as an introduction to a complex and, at present, incompletely unravelled problem. The new chapter on the Electroencephalogram will prove helpful and easy to follow. Altogether, this is an extraordinarily useful little compendium for busy practitioners and for students.

